# Beyond counting sheep: social and behavioral determinants of adolescent sleep quality in the Czech Republic

**DOI:** 10.1186/s12889-025-26052-2

**Published:** 2026-01-30

**Authors:** Katerina Veskrnova, Jana Furstova, Peter Tavel

**Affiliations:** Olomouc University Social Health Institute, Sts Cyril and Methodius Faculty of Theology, Palacký University Olomouc, Univerzitní 244/22, 77900 Olomouc, Czech Republic

**Keywords:** Sleep quality, Adolescents, HBSC study, Social support, Psychosocial factors, Health behaviors, Czech republic

## Abstract

**Background:**

Adolescence is a critical period of neurodevelopment, during which adequate sleep is essential for cognitive, emotional, and physical health. Despite extensive research on sleep duration, there is limited data on the social, behavioral, and psychosocial determinants of sleep quality among Czech adolescents. This study aims to investigate these factors and their associations with adolescent sleep quality.

**Methods:**

Using a sample of 4,508 adolescents aged 13–15 years from the 2021/22 HBSC survey, sleep quality was assessed using the Short Adolescent Sleep Wake Scale (ASWS). Sociodemographic variables, social environment, stressors, health and health behaviors were assessed. Multivariate linear regression models were employed to identify significant links with sleep quality.

**Results:**

In multivariate analyses, clinically meaningful associations (≥ 0.2 SD in absolute value) were observed for sex and sleep duration, with girls showing lower overall sleep quality (non-standardized b = -2.1, 95% CI [-2.6, -1.6]) and adolescents reporting longer sleep showing higher overall sleep quality (β = 1.8, 95% CI [1.6, 2.1]). Smaller but consistent associations (≥ 0.1 SD in absolute value) were observed for age (non-standardized b = 1.5, 95% CI [1.0, 2.0]), overall health (β = 1.3, 95% CI [1.1, 1.6]), academic pressure (β = -1.7, 95% CI [-1.9, -1.5]), and perceived family support (β = 1.3, 95% CI [1.0, 1.6]). No meaningful associations were observed between overall sleep quality and either friend support or physical activity.

**Conclusions:**

These results highlight the multifaceted nature of adolescent sleep quality and emphasize the associations with psychosocial and behavioral factors. Although no single factor showed a large clinical effect, their cumulative influence may be meaningful. Interventions targeting multiple modest-risk factors simultaneously – such as reducing academic pressure, increasing family support, and limiting screen time – could improve adolescent sleep quality at the population level.

**Supplementary Information:**

The online version contains supplementary material available at 10.1186/s12889-025-26052-2.

## Background

Humans spend about a third of their lives sleeping. Sleep therefore represents an essential element in our lives and has been recognized as a foundation for health and well-being [1]. During adolescence, adequate sleep is critically important, primarily due to the substantial neurodevelopmental changes that take place during this period [2]. Adequate sleep has been associated with better cognitive functions, including memory, attention and executive functioning, all of which are essential for learning and academic performance [3, 4]. Further, sleep is linked to emotional regulation, contributing to better mental health and overall well-being [5]. On the other hand, poor-quality sleep has been connected to poorer cognitive functions such as attention, problem-solving, and memory consolidation [6–8]. Prolonged sleep deprivation has been associated with an elevated risk of psychological disorders, including depression, anxiety, and mood dysregulation [5, 9]. Adequate sleep length is also vital for maintaining physical health [5, 10]. Adolescents who do not get enough sleep may experience disruptions in growth or impairments in immune function, leading to an increased susceptibility to illness [7]. Insufficient sleep has been associated with reduced function of the prefrontal cortex, a region critical for impulse control, as well as with greater susceptibility to engage in risky behaviors such as substance use [5, 11, 12].

Both the quantity and quality of sleep are essential components of healthy sleep in adolescence. In terms of sleep quantity, it is recommended that children aged 6–12 years achieve 9 to 12 h of sleep, while adolescents aged 13–17 years should aim for 8 to 10 h [1]. The assessment of sleep quality, however, requires a more complex approach. In general, sleep quality describes the extent to which sleep is restorative and free from interruptions [13]. Sleep quality is commonly evaluated across four key dimensions [13]: (1) sleep latency, measuring the time required to fall asleep; (2) awakenings, assessing the frequency of nighttime wakefulness; (3) wake after sleep onset, indicating the total time spent awake after initially falling asleep; and (4) sleep efficiency, representing the proportion of time spent asleep relative to total time in bed.

If the quality of sleep is poor, adolescents may not adequately experience the restorative stages of sleep, even if they reach the recommended sleep duration. As a result, sleep quality may be a more comprehensive indicator of sleep health than sleep duration alone [10, 14, 15]. Several factors have been associated with variations in sleep quality, including sleep timing and sleep hygiene [16]. Adolescence is characterized by a pronounced shift in the endogenous circadian rhythm, reflecting alterations in sleep-wake regulatory mechanisms [17–20]. This misalignment can result in difficulties falling asleep early enough to get high-quality sleep. To address these challenges, existing recommendations emphasize lifestyle practices associated with better sleep hygiene. These include engaging in regular physical activity [21–23], limiting consumption of caffeine [24–26], avoiding nicotine use [27], and maintaining healthy dietary patterns [28]. Additionally, managing screen time [29–31], particularly by restricting social media use before bedtime, has been shown to contribute to better sleep quality [32].

Sleep quality can be assessed using both objective and subjective methods. Objective measures, such as polysomnography conducted in laboratory settings or wearable electronic devices (e.g. watches, rings, or headbands) provide precise biological data on sleep patterns. In contrast, subjective assessment methods rely on self-report tools, capturing individuals’ perceptions of their sleep and related behaviors, which may not be fully reflected in objective data [33]. Several validated instruments are available for adolescent sleep assessment, e.g. the Pittsburgh Sleep Quality Index (PSQI) [34–36], and the short Adolescent Sleep Wake Scale (ASWS) [37, 38]. The ASWS, in particular, is widely recognized and frequently used for assessing sleep quality in adolescent populations [37]. Previous research indicates that subjective assessment tools are particularly effective in evaluating psychological factors of perceived sleep quality, which are not fully captured by objective metrics (Yetton B, McDuff D, Barakat A, Jiang A, Allen NB, Schneider LD, et al: Perspective chapter: assessment of subjective and objective sleep quality from Wrist-Worn wearable data, forthcoming).

In the context of adolescent sleep behaviors, the Czech Republic ranks among the countries with the shortest average sleep duration, with fewer than 50% of Czech adolescents meeting the CDC sleep recommendations on school days [9]. Despite this concerning trend, to the best of our knowledge, no large-scale study has specifically investigated the sleep behaviors of Czech adolescents with an emphasis on sleep quality, considering the interplay of biological, psychological, and social factors. Recent literature emphasizes the importance of conceptualizing adolescent sleep within a biopsychosocial and contextual framework, which integrates biological processes (e.g., pubertal and neurodevelopmental changes), psychological mechanisms (e.g., stress, affect regulation), and social-contextual influences (e.g., family, school, and peer environments) [39, 40]. Guided by this framework, the present study aims to address the existing gap by investigating the differences in sleep quality among sociodemographic groups, and examining the associations between biological (e.g., age, sex), psychological (e.g. stress, bullying), social (e.g. perceived social support), and behavioral factors and sleep quality among Czech adolescents. By identifying key correlates of sleep quality within this multidimensional framework, the study seeks to provide insights that may inform targeted interventions and public health policies to support adolescent sleep health. Understanding these relationships is essential for developing strategies to reduce poor sleep quality and its associated negative consequences.

## Methods

### Data and participants

This study used data collected within the framework of the Health Behavior in School-aged Children (HBSC) study, an international cross-sectional survey conducted in collaboration with the World Health Organization (http://www.hbsc.org). The HBSC surveys, conducted every four years, examine health, lifestyle and their social determinants among 11-, 13-, and 15-year-old students. The present study utilized data from the 2021/22 wave of the HBSC study in the Czech Republic. The Czech HBSC dataset fully adheres to the international HBSC study protocol [41].

#### Inclusion criteria

The study included nationally representative data from students enrolled in the 7th and 9th grades across 242 schools in all 14 administrative regions of the Czech Republic. Participants were included if they completed the version of the HBSC questionnaire containing items on sleep quality. The final analytic sample comprised 4,508 adolescents (mean age 14.6 ± 1.1years, 48.6% girls).

#### Exclusion criteria

Participants with missing data on key study variables were excluded from the analyses. The participant flow, including exclusions, is presented in Supplementary Figure S1.

#### Ethical approval

Participation in the survey was anonymous and voluntary, with no monetary or other incentives provided. Informed consent was obtained either directly from participants’ parents/guardians or indirectly from school principals, in cases where parents had provided “general informed consent” at the beginning of the school year. This general consent allowed students to participate in surveys and other school activities organized during the academic year. The study was conducted in accordance with the principles of the Declaration of Helsinki and was approved by the ethics committee of the Faculty of Physical Culture, Palacký University Olomouc (No. 14/2019).

### Design

The design of this study was cross-sectional. The outcome variables examined were the overall quality of sleep in adolescents and its three components: the process of going to bed and falling asleep, the ability to maintain or reinitiate sleep, and the transition to wakefulness. Guided by the biopsychosocial model, the primary focus of the study was to examine the associations between adolescent sleep quality and factors spanning biological (age, sex, health), psychological (stressors, bullying), and social (family, peer, and school environment) domains, as well as health behaviors.

### Variables in the study

#### Quality of sleep

The short Adolescent Sleep Wake Scale (short ASWS) [38] was used to measure the overall quality of sleep in adolescents. The short ASWS is a 10-item questionnaire with three subscales assessing bedtime behaviors (5 items, α = 0.66), sleep efficiency (3 items, α = 0.78), and morning wakefulness (2 items, α = 0.88). A summary score of the overall quality of sleep (0–50) as well as summary scores of the subscales are used, with higher summary scores corresponding to higher sleep quality. Although no formal MID exists for the 10-item ASWS (0–50), a change of ≥ 5 points is considered a conservative threshold for minimal clinical relevance, based on effect-size mapping from the scale’s development [37] and clinical use of the full ASWS [42, 43]. A 1-point change thus represents 20% of this threshold. To aid interpretability and comparability, we also fitted standardized outcome models (with ASWS z-standardized) and present them in the Supplement.

#### Sociodemographic and biological variables

Participants were asked to report their sex (boy/girl) and month and year of birth, which were used to determine their age group (13 or 15 years, corresponding to 7th and 9th grades, respectively). For estimation of the socio-economic status (SES) of the participants’ families, the Family Affluence Scale (FAS) was used. FAS is a valid and reliable scale [44] used within the HBSC network. It consists of six items assessing number of cars, computers, bathrooms, and family holidays spent abroad, ownership of a dishwasher, and having own bedroom, during the past 12 months. *Self-rated health* was assessed with a single item “Would you say your health is”, with four response options from “excellent” to “poor”. The coding of this item has been reversed before the analyses so that higher number corresponded to better health. Age, sex, health and SES are considered biological and structural determinants that may be linked to adolescent sleep.

#### Psychological variables/stressors

Bullying, cyberbullying, and academic pressure were treated as psychosocial stressors, in line with the psychological domain of the biopsychosocial model. Bullying and cyberbullying victimization, along with academic pressure, were evaluated as sources of stress. To assess bullying and cyberbullying victimization, questions adapted from the Olweus Bully/Victim Questionnaire [45] were used: “How often have you been bullied at school in the past couple of months?” and “In the past couple of months how often have you been cyberbullied?”. Both questions were answered on a 5-point scale from “I have not been bullied/cyberbullied” to “several times per week”. The feeling of being pressured by the demands of schoolwork was measured with a single item “How pressured do you feel by the schoolwork you have to do?”, with possible answers on a 4-point scale from “not at all” to “a lot”.

#### Social environment variables

Family, teacher and student support were considered social determinants of sleep quality, reflecting the social domain of the model. *The Multidimensional Scale of Perceived Social Support* [46] was used to assess perceived social support from family and friends. The corresponding subscales consist of four items assessing family support (α = 0.93) and four items assessing friend support (α = 0.93). Each item was rated on a seven-point Likert scale. Perceived social support from teachers and students was assessed with the *Teacher and Classmate Support Scale* [47]. The corresponding subscales contain three items each (α = 0.80 for both teacher and student support). The items were rated on a five-point Likert scale. Higher scores on the family, friend, teacher, and student support subscales indicate greater perceived social support.

#### Health behaviors

Physical activity, substance use, and screen time were included as behavioral factors, complementing the biological, psychological, and social determinants in the model. *Health behaviors* were assessed by collecting self-reported data on participants’ average length of sleep on schooldays, substance use (alcohol and cigarettes), energy drink consumption, physical activity levels, and screen time. Alcohol consumption and smoking cigarettes were evaluated by asking “On how many days (if any) have you drunk alcohol/smoked cigarettes in the last 30 days?“ [48]. The possible answers ranged from “Never” to “30 days (or more)”. Participants were asked “How many times a week do you usually drink energy drinks?”, with possible answers on a 7-point scale from “never” to “every day, more than once”. Daily moderate-to-vigorous physical activity (MVPA) was measured with an item “Over the past 7 days, on how many days were you physically active for a total of at least 60 minutes per day? Please add up all the time you spent in physical activity each day.“ [49]. The possible answers were 0 to 7 days. Screen time behavior was assessed through questions regarding the average number of hours spent per day, during free time, on (1) playing games on electronic devices, (2) engaging in social media communication, (3) watching television or videos, and (4) browsing the internet for information or other purposes. There were nine response options to each of the questions, from “none at all” to “seven or more hours a day”. The answers were treated as continuous (in hours).

### Statistical analysis

#### Missing data

Participants with missing data on the outcome variables (sleep quality or its subscales) were excluded from the analyses (*n* = 589). Missing values among independent variables ranged from 0.1% to 8.3% and were assumed to be missing at random (MAR). To minimize data loss and potential bias, missing independent variable values were imputed using multiple imputation by chained equations (MICE; five imputations) in R (mice package). All independent variables were included in the imputation model to preserve their mutual associations. Outcome variables were not imputed and were excluded as predictors from the imputation procedure. The proportion of missing values for each variable is shown in Table 2, and the participant flow is presented in Supplementary Figure S1.

#### Descriptive and regression analyses

Descriptive characteristics of the study sample were computed and sleep quality was compared across sex and age groups, using the Welch test for unequal variances. In addition to the *p*-values, effect size coefficients (Cohen’s d) are presented.

Associations between sleep quality and its subscales (bedtime behaviors, sleep efficiency, and morning wakefulness) and the biological, psychological, social and behavioral factors were examined using multivariate linear regression models. Independent variables were organized according to the biopsychosocial framework, and the hypothesized relations are depicted in Supplementary Figure S2. All continuous independent variables in the models were mean-centered (for estimating b coefficients) and standardized (for estimating β coefficients) to allow for comparison. As a sensitivity analysis, the models were re-estimated using complete cases only. The imputed and complete-case results were consistent in direction, magnitude, and significance, indicating that imputation did not materially affect the conclusions. The complete-case models are presented in Supplementary Table S3. To assess the practical and clinical relevance of the independent variables, fully standardized models were estimated, in which both independent and dependent variables were standardized with z-scores (see Supplementary Table S4). Fully standardized estimates (Stdβ) are provided to facilitate interpretation of effect sizes. Following recommendations for patient-reported outcomes when no anchor-based MID is available [50, 51], standardized effects of 0.2 SD, 0.5 SD and 0.8 SD were considered as small, moderate and large, respectively.

All the statistical analyses were performed in the R software, version 4.3.0 (R Core Team, 2023) using the RStudio environment, version 2024.04.2. All analyses were conducted using the Survey package in R [52] to account for survey data with appropriate weights.

## Results

### Descriptive characteristics

**Fig. 1:**
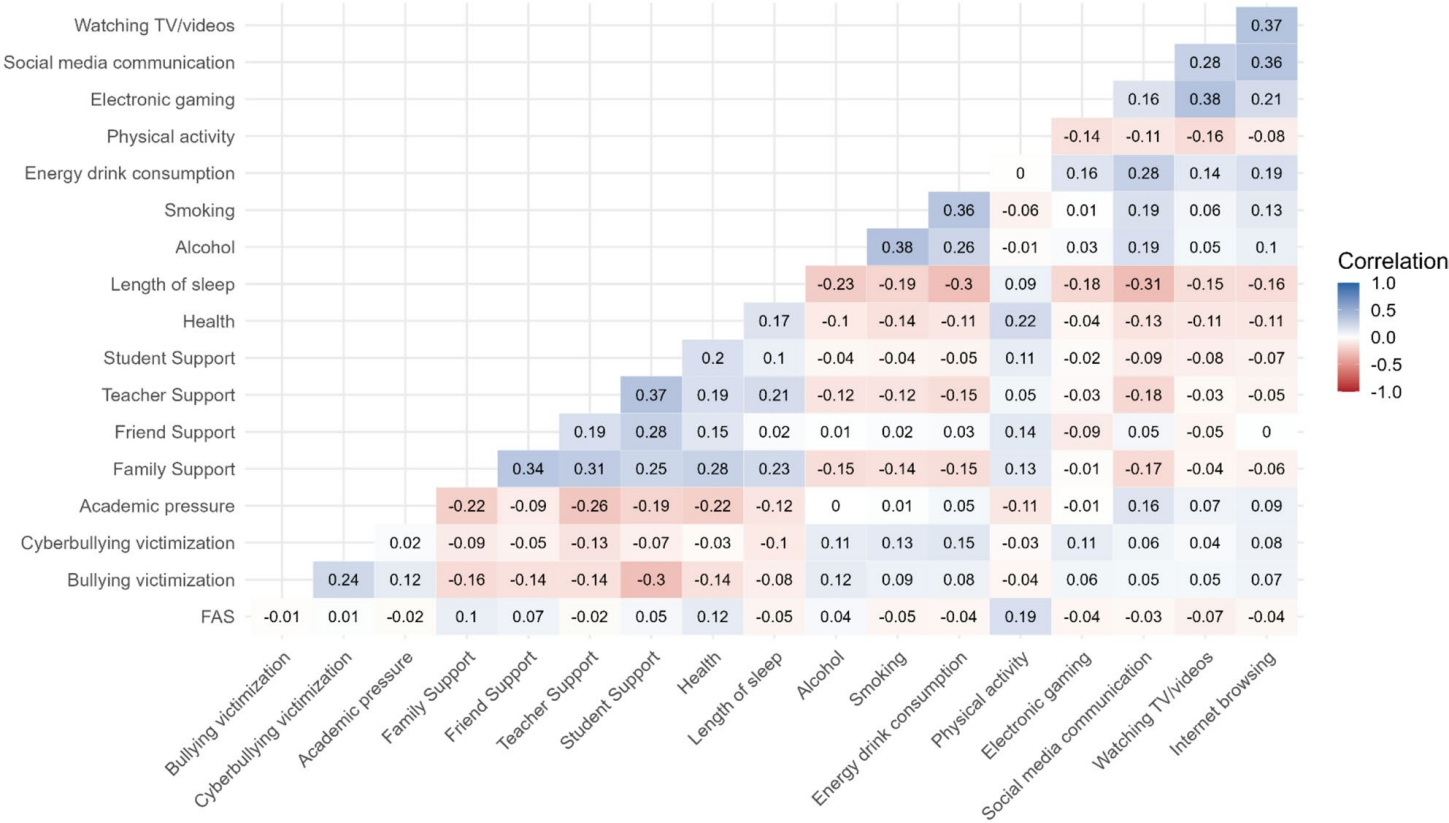
Lower-triangle correlation heatmap of continuous study variables. Health Behaviour in School-aged Children study, Czech Republic, 2022

Tables 1 and 2 present the descriptive characteristics of the study sample and the comparison of the quality of sleep across sex and age groups. Girls reported lower overall sleep quality and lower scores on the sleep efficiency and morning wakefulness subscales compared to boys, with moderate effect sizes (Cohen’s d: 0.36─0.44). No meaningful differences were observed in bedtime behaviors between girls and boys. Older adolescents exhibited slightly higher sleep efficiency but lower morning wakefulness compared to younger adolescents, both with small effect sizes (Cohen’s d ≤ 0.1). Intercorrelations among the continuous independent variables used in subsequent regression models are presented in a heatmap (Fig. 1). Correlations were generally low, with absolute values ranging from 0 to 0.38, indicating limited overlap between the independent variables.

**Table 1:** Descriptive characteristics of the study sample and comparison of sleep quality between the sex and age groups. Health behaviour in School-aged children study, Czech Republic, 2022

Variable	*n* (%)	Overall Sleep Quality Mean (SD)	Bedtime Behaviors Mean (SD)	Sleep Efficiency Mean (SD)	Morning Wakefulness Mean (SD)
Overall	4508 (100)	31.3 (8.70)	7.8 (3.19)	19.4 (5.60)	4.0 (2.80)
Sex †					
Boys	2317 (51.4)	32.9 (8.1)	7.9 (3.2)	20.4 (5.1)	4.6 (2.8)
Girls	2191 (48.6)	29.5 (9.0)	7.7 (3.2)	18.4 (5.9)	3.4 (2.6)
P-value		< 0.001	0.016	< 0.001	< 0.001
Effect size (Cohen d)		0.40	0.07	0.36	0.44
Age category †					
13 years	2034 (45.1)	31.0 (9.1)	7.8 (3.3)	19.1 (5.7)	4.2 (2.9)
15 years	2474 (54.9)	31.5 (8.4)	7.9 (3.1)	19.7 (5.5)	3.9 (2.7)
*P*-value		0.09	0.36	< 0.001	0.007
Effect size (Cohen d)		0.05	0.03	0.10	0.08

*SD* standard deviation, † *P*-values obtained from the Welch test comparing means between two groups with unequal variances

**Table 2:** Descriptive characteristics for continuous dependent and independent variables among adolescents (*n* = 4,508). Health behaviour in School-aged children study, Czech Republic, 2022

Dependent variables	Mean (SD)	Min–Max	% missing	Interpretation
*Sleep Quality*				
Overall Sleep Quality	31.26 (8.70)	0 ─ 50	0.0	Higher summary score = higher sleep quality.
Bedtime Behaviors	7.81 (3.19)	0 ─ 15	0.0	Higher summary score = higher sleep quality.
Sleep Efficiency	19.41 (5.60)	0 ─ 25	0.0	Higher summary score = higher sleep quality.
Morning Wakefulness	4.04 (2.80)	0 ─ 10	0.0	Higher summary score = higher sleep quality.
Independent variables				
*Socio-demographic and biological variables*				
FAS	8.37 (2.25)	0 ─ 13	1.5	Higher summary score = higher SES.
Health	3.10 (0.72)	1 ─ 4	0.6	Higher score = better health.
*Stressors*				
Bullying victimization	1.36 (0.87)	1 ─ 5	7.1	Higher score = more frequent.
Cyberbullying victimization	1.15 (0.57)	1 ─ 5	8.3	Higher score = more frequent.
Academic pressure	2.61 (0.95)	1 ─ 4	0.1	Higher score = more stress.
*Social environment*				
Family Support	5.38 (1.70)	1 ─ 7	3.5	Higher summary score = higher social support.
Friend Support	5.22 (1.64)	1 ─ 7	4.2	Higher summary score = higher social support.
Teacher Support	2.23 (0.92)	0 ─ 4	0.2	Higher summary score = higher social support.
Student Support	2.36 (0.92)	0 ─ 4	0.4	Higher summary score = higher social support.
*Health behaviors*				
Length of sleep [hours]	7.65 (1.06)	4 ─ 10	1.3	Length in hours.
Alcohol	1.61 (1.10)	1 ─ 7	3.0	Higher score = more frequent.
Smoking	1.31 (1.13)	1 ─ 7	2.1	Higher score = more frequent.
Energy drink consumption	1.99 (1.54)	1 ─ 7	0.3	Higher score = more frequent.
Physical activity at least 60 min	4.30 (2.01)	0 ─ 7	0.3	Higher score = more frequent.
Electronic gaming [hours]	2.13 (2.10)	0 ─ 7	0.7	Length in hours.
Social media communication [hours]	2.67 (2.13)	0 ─ 7	0.9	Length in hours.
Watching TV/videos [hours]	1.96 (1.76)	0 ─ 7	1.0	Length in hours.
Internet browsing [hours]	1.18 (1.53)	0 ─ 7	0.9	Length in hours.

### Determinants of sleep quality

Table 3 presents the results of multivariate regression analyses examining associations between biological, psychological, social, and behavioral determinants and adolescent sleep quality. Clinically meaningful effects, defined as small effects exceeding 0.2 SD (in absolute value) in the fully standardized models (Supplementary Table S4), were observed for sex and sleep duration. Smaller but consistent effects (≥ 0.1 SD in absolute value) were found for several other factors, including age, overall health, academic pressure, perceived social support, and screen time.

**Table 3:** Summary of the regression analysis; Overall sleep quality and its related subscales, i.e. bedtime behaviors, sleep efficiency, and morning wakefulness (dependent variables) in association with biological, psychological, social, and behavioral factors (independent variables); Health Behaviour in School-aged Children study, Czech Republic, 2022

Independent variables	Overall Sleep Quality					Bedtime Behaviors		
	b (95% CI)		β (95% CI)		*p*-value	b (95% CI)	β (95% CI)	*p*-value
*Socio-demographic and biological variables*								
Sex [Girls vs Boys]	−2.08 (−2.57, −1.58)				<0.001	−0.12 (−0.32, 0.07)		0.215
Age [15 vs 13 years]	1.48 (0.98, 1.97)				<0.001	0.51 (0.33, 0.69)		<0.001
FAS	0.01 (−0.09, 0.10)		0.02 (−0.19, 0.23)		0.870	0.00 (−0.04, 0.04)	0.00 (−0.09, 0.09)	0.977
Health	1.81 (1.46, 2.15)		1.31 (1.05, 1.56)		<0.001	0.30 (0.15, 0.45)	0.22 (0.11, 0.33)	<0.001
*Stressors*								
Bullying victimization	−0.31 (−0.59, −0.03)		−0.27 (−0.52, −0.03)		0.031	0.01 (−0.11, 0.13)	0.01 (−0.09, 0.11)	0.846
Cyberbullying victimization	−0.20 (−0.57, 0.18)		−0.11 (−0.33, 0.10)		0.306	−0.01 (−0.18, 0.16)	−0.01 (−0.10, 0.09)	0.890
Academic pressure	−1.78 (−2.02, −1.54)		−1.68 (−1.91, −1.46)		<0.001	−0.25 (−0.36, −0.14)	−0.24 (−0.34, −0.14)	<0.001
*Social environment*								
Family Support	0.78 (0.61, 0.94)		1.32 (1.04, 1.60)		<0.001	0.18 (0.12, 0.25)	0.31 (0.21, 0.42)	<0.001
Friend Support	0.05 (−0.11, 0.20)		0.07 (−0.17, 0.32)		0.555	−0.02 (−0.08, 0.04)	−0.04 (−0.13, 0.06)	0.451
Teacher Support	0.48 (0.21, 0.75)		0.44 (0.20, 0.69)		<0.001	0.10 (−0.02, 0.21)	0.09 (−0.02, 0.19)	0.093
Student Support	0.69 (0.36, 1.01)		0.63 (0.33, 0.94)		<0.001	0.09 (−0.04, 0.21)	0.08 (−0.03, 0.19)	0.173
*Health behaviors*								
Length of sleep	1.70 (1.45, 1.95)		1.81 (1.55, 2.08)		<0.001	0.83 (0.73, 0.92)	0.88 (0.78, 0.98)	<0.001
Alcohol	−0.38 (−0.61, −0.15)		−0.42 (−0.67, −0.16)		0.001	−0.13 (−0.22, −0.04)	−0.14 (−0.24, −0.04)	0.006
Smoking	0.09 (−0.13, 0.32)		0.11 (−0.15, 0.36)		0.404	0.13 (0.05, 0.22)	0.15 (0.05, 0.25)	0.003
Energy drink consumption	−0.48 (−0.67, −0.30)		−0.74 (−1.03, −0.46)		<0.001	−0.07 (−0.14, 0.00)	−0.10 (−0.21, 0.01)	0.067
Physical activity at least 60 min	0.04 (−0.08, 0.15)		0.07 (−0.16, 0.30)		0.537	0.03 (−0.02, 0.08)	0.06 (−0.03, 0.16)	0.187
Electronic gaming	−0.32 (−0.45, −0.19)		−0.66 (−0.94, −0.39)		<0.001	−0.18 (−0.23, −0.13)	−0.37 (−0.47, −0.27)	<0.001
Social media communication	−0.23 (−0.35, −0.10)		−0.49 (−0.76, −0.22)		<0.001	−0.09 (−0.14, −0.04)	−0.19 (−0.30, −0.08)	0.001
Watching TV/videos	−0.16 (−0.31, −0.01)		−0.28 (−0.55, −0.01)		0.039	−0.09 (−0.15, −0.03)	−0.16 (−0.26, −0.06)	0.003
Internet browsing	−0.15 (−0.33, 0.04)		−0.22 (−0.51, 0.06)		0.123	0.06 (0.00, 0.13)	0.10 (−0.01, 0.20)	0.068
R^2^	0.389					0.210		

Independent variables	Sleep Efficiency					Morning Wakefulness		
	b (95% CI)	β (95% CI)		*p*-value		b (95% CI)	β (95% CI)	*p*-value
*Socio-demographic and biological variables*								
Sex [Girls vs Boys]	−1.30 (−1.67, −0.93)			<0.001		−0.65 (−0.83, −0.47)		<0.001
Age [15 vs 13 years]	1.07 (0.73, 1.40)			<0.001		−0.10 (−0.27, 0.06)		0.229
FAS	0.04 (−0.03, 0.11)	0.09 (−0.07, 0.24)		0.268		−0.03 (−0.07, 0.00)	−0.07 (−0.15, 0.01)	0.077
Health	1.01 (0.77, 1.24)	0.73 (0.56, 0.90)		<0.001		0.50 (0.39, 0.61)	0.36 (0.28, 0.44)	<0.001
*Stressors*								
Bullying victimization	−0.37 (−0.59, −0.16)	−0.33 (−0.52, −0.14)		0.001		0.05 (−0.04, 0.14)	0.04 (−0.03, 0.12)	0.265
Cyberbullying victimization	−0.14 (−0.43, 0.15)	−0.08 (−0.25, 0.09)		0.342		−0.04 (−0.17, 0.08)	−0.03 (−0.10, 0.05)	0.483
Academic pressure	−1.07 (−1.24, −0.90)	−1.01 (−1.17, −0.85)		<0.001		−0.46 (−0.55, −0.37)	−0.44 (−0.52, −0.35)	<0.001
*Social environment*								
Family Support	0.36 (0.24, 0.47)	0.61 (0.41, 0.80)		<0.001		0.24 (0.19, 0.29)	0.41 (0.32, 0.49)	<0.001
Friend Support	0.00 (−0.11, 0.11)	−0.01 (−0.19, 0.17)		0.940		0.07 (0.02, 0.12)	0.12 (0.04, 0.20)	0.004
Teacher Support	0.01 (−0.20, 0.21)	0.01 (−0.18, 0.19)		0.946		0.38 (0.27, 0.48)	0.35 (0.25, 0.44)	<0.001
Student Support	0.32 (0.09, 0.55)	0.30 (0.09, 0.51)		0.006		0.28 (0.18, 0.37)	0.26 (0.17, 0.35)	<0.001
*Health behaviors*								
Length of sleep	0.66 (0.47, 0.85)	0.70 (0.50, 0.90)		<0.001		0.21 (0.14, 0.29)	0.23 (0.14, 0.31)	<0.001
Alcohol	−0.22 (−0.39, −0.05)	−0.25 (−0.44, −0.06)		0.010		−0.02 (−0.10, 0.05)	−0.03 (−0.11, 0.06)	0.524
Smoking	−0.05 (−0.24, 0.14)	−0.06 (−0.27, 0.16)		0.599		0.01 (−0.06, 0.08)	0.02 (−0.06, 0.10)	0.705
Energy drink consumption	−0.38 (−0.51, −0.24)	−0.58 (−0.79, −0.37)		<0.001		−0.04 (−0.10, 0.02)	−0.06 (−0.15, 0.02)	0.153
Physical activity at least 60 min	−0.09 (−0.17, 0.00)	−0.17 (−0.34, −0.01)		0.043		0.09 (0.05, 0.13)	0.18 (0.11, 0.26)	<0.001
Electronic gaming	−0.09 (−0.19, 0.01)	−0.19 (−0.39, 0.02)		0.070		−0.05 (−0.09, −0.01)	−0.11 (−0.19, −0.02)	0.017
Social media communication	−0.01 (−0.11, 0.09)	−0.02 (−0.23, 0.18)		0.843		−0.13 (−0.17, −0.09)	−0.28 (−0.36, −0.19)	<0.001
Watching TV/videos	−0.04 (−0.15, 0.07)	−0.08 (−0.27, 0.12)		0.432		−0.03 (−0.08, 0.02)	−0.05 (−0.14, 0.04)	0.285
Internet browsing	−0.36 (−0.50, −0.21)	−0.55 (−0.77, −0.32)		<0.001		0.15 (0.09, 0.20)	0.22 (0.14, 0.30)	<0.001
R^2^	0.254					0.293		

Continuous independent variables were mean centered and standardized to compute b and β coefficients, respectively. For categorical variables, only b coefficients are shown, as standardization is not applicable. The variation inflation factors (VIF) ≤ 1.66 for all variables in all models, i.e. no multicollinearity was detected

Specifically, negative associations with overall quality of sleep were observed for sex (non-standardized b=−2.1, 95% CI [−2.6, −1.6]) and academic pressure (β=−1.7, 95% CI [−1.9, −1.5]), indicating that girls and adolescents reporting higher academic stress tended to have lower sleep quality. In contrast, higher age (non-standardized b = 1.5, 95% CI [1.0, 2.0]), better overall health (β = 1.3, 95% CI [1.1, 1.6]), higher perceived family support (β = 1.3, 95% CI [1.0, 1.6]), and longer sleep duration (β = 1.8, 95% CI [1.6, 2.1]) were positively associated with overall sleep quality.

For the bedtime behaviors subscale, the only clinically relevant association (≥ 0.2 SD) was observed with the duration of sleep (β = 0.9, 95% CI [0.8, 1.0]). Other positive associations with smaller effect were observed for age (non-standardized b = 0.5, 95% CI [0.3, 0.7]), and family support (β = 0.3, 95% CI [0.2, 0.4]). A negative association was found only for time spent on electronic gaming (β=−0.4, 95% CI [−0.5, −0.3]).

Sleep efficiency exhibited a clinically relevant (≥ 0.2 SD) negative association with sex (non-standardized b=−1.3, 95% CI [−1.7, −0.9]). Smaller negative effects (≥ 0.1 SD) were found with academic pressure (β=−1.0, 95% CI [−1.2, −0.9]), energy drink consumption (β=−0.6, 95% CI [−0.8, −0.4]), or time spent browsing the internet (β=−0.6, 95% CI [−0.8, −0.3]). Positive associations with smaller effect (≥ 0.1 SD) were observed for older age (non-standardized b = 1.1, 95% CI [0.7, 1.4]), better overall health (β = 0.7, 95% CI [0.6, 0.9]), perceived family support (β = 0.6, 95% CI [0.4, 0.8]), and sleep duration (β = 0.7, 95% CI [0.5, 0.9]).

Finally, the morning wakefulness subscale was negatively associated with sex (non-standardized b=−0.7, 95% CI [−0.8, −0.5]), academic pressure (β=−0.4, 95% CI [−0.5, −0.4]), or time spent on social media (β=−0.3, 95% CI [−0.4, −0.2]). Positive associations were observed for overall health (β = 0.4, 95% CI [0.3, 0.4]) and perceived social support from family (β = 0.4, 95% CI [0.3, 0.5]) and teachers (β = 0.4, 95% CI [0.3, 0.4]).

## Discussion

The present study aimed to evaluate the sleep quality of Czech adolescents and examine how biological (e.g., age, sex, health), psychological (e.g., academic pressure, bullying), social (e.g., family, teacher and student support), and behavioral factors (e.g., sleep duration, substance use, screen time) are jointly associated with sleep quality. Clinically meaningful associations were observed primarily for sex and sleep duration, with smaller but consistent effects for age, overall health, academic pressure, family support, and screen time. These results support the biopsychosocial perspective on adolescent sleep health, suggesting that psychosocial and environmental contexts – such as family support, academic demands, and lifestyle behaviors –may exert equal or greater influence on sleep quality than biological factors alone.

Our findings are consistent with a recent international HBSC analysis across 24 countries and more than 165,000 adolescents [9], which reported substantial cross-national variations in sleep duration and timing, as well as pervasive irregularities in sleep patterns known as social jetlag. The Czech data extend this work by demonstrating particularly pronounced gender disparities and the strong link between sleep duration and overall sleep quality, alongside the roles of psychosocial stress and family support. Together, these findings underscore the importance of considering country-specific educational and cultural contexts when developing interventions to promote adolescent sleep health.

Sex differences were among the most consistent findings: girls reported poorer overall sleep quality, sleep efficiency and morning wakefulness compared to boys. These findings are consistent with previous research [53, 54], although some studies have found no significant gender differences [55]. The underlying mechanisms explaining these differences are not yet fully understood. Several hypotheses have been proposed, including hormonal changes related to puberty and menstrual cycles that may influence sleep patterns and quality [56, 57]. Psychological factors may also play a role, as adolescent girls tend to report higher levels of stress, anxiety and depressive symptoms, which are known to interfere with sleep quality [58]. Social dynamics may also play a role as girls tend to be more emotionally and psychologically engaged in peer relationships, and negative interpersonal experiences can put more strain on their mental health compared to boys [59, 60]. Additionally, research indicates that boys and girls utilize social media differently [60], with girls being more vulnerable to its negative effects [60]. Girls often use social media in ways that may promote social comparison and rumination, which can delay sleep onset and further impair their sleep quality [61, 62]. These biological, psychosocial, and behavioral factors, likely interact and contribute to the lower sleep quality reported among adolescent girls.

Older adolescents reported better overall sleep quality, which may reflect increased autonomy in managing sleep routines, improved emotion regulation skills, and greater resilience to sleep disturbances [63]. While younger adolescents often face a developmental transition that disrupts established sleep patterns, they also tend to be more sensitive to environmental stressors [64]. Biological changes, such as delayed circadian rhythms during puberty, can cause a shift toward “eveningness” [56, 64], which clashes with early school start times and contributes to sleep deficits. Younger adolescents also have less control over their sleep environment and bedtime routines [65, 66], and their coping mechanisms for managing stress are still developing [7]. These factors may compound to create lower sleep quality among younger adolescents, despite fewer academic and social obligations compared to older peers.

In this study, there was a very weak association between socioeconomic status (SES) and sleep quality. While some previous research has reported associations between lower SES and poorer sleep quality or shorter sleep duration [64, 67–69], our findings are consistent with the international HBSC study [9], which showed that SES–sleep associations varied across cultural and policy contexts. These associations tend to be stronger in countries with greater socioeconomic inequalities or less comprehensive welfare systems. The Czech Republic may represent a distinctive case in this regard. As one of Europe’s more egalitarian societies, with a state-supported education system, universal healthcare, and relatively low income inequality [70], the material and psychosocial stressors typically linked to socioeconomic disadvantage may be partially buffered.

Among health-related variables, sleep duration and self-rated health showed robust positive associations with sleep quality. These findings reinforce the importance of holistic health promotion strategies. Consistent with previous European HBSC findings [71] and other international evidence [72], adequate sleep duration has been identified as one of the strongest and most consistent predictors of self-rated health and also as health-related quality of life among adolescents. These findings indicate that inadequate sleep duration are associated with poorer perceived health, greater psychological distress, and lower overall well-being [71, 72]. Conversely, maintaining the recommended sleep duration for adolescents, as defined by the CDC [1], is linked to better emotional regulation, cognitive functioning, and overall life satisfaction.

Smaller but consistent associations were found for family support and academic pressure. Among all sources of support, family support demonstrated the strongest positive association with overall sleep quality and with two of the subscales: bedtime behaviors and sleep efficiency. Our findings are consistent with previous studies [73, 74]. A supportive family environment is likely associated with a greater sense of safety and stability, which may be linked to lower stress and anxiety and more consistent routines, potentially supporting better sleep hygiene [75]. Morning wakefulness, a distinct subscale, was positively associated with all four types of social support: from family, friends, teachers, and students. This underscores the potential importance of a nurturing social environment for adolescents’ alertness and well-being during the day. Interestingly, friend support was not associated with overall sleep quality [73, 76]. Weaker peer effects may reflect developmental or cultural differences in how social connectedness influences sleep in early versus mid-adolescence. This finding warrants further exploration, as peer relationships are often considered crucial during adolescence [77].

In this study, family support had a stronger association with sleep quality than physical activity. This contrasts with some previous research that has shown positive effects of physical activity on adolescent sleep quality [54, 78, 79]. While physical activity is undoubtedly beneficial, our findings suggest that the emotional support provided within the family may play an even more essential role in promoting healthy sleep. As expressed in the idea that “a hug is more than sport” [80], emotional closeness and psychological safety may be especially vital for adolescent well-being. This message has important implications not only for professionals but also for families, educators, and society more broadly.

As a main stressor at the psychological level, academic pressure showed consistent negative associations with overall sleep quality, sleep efficiency and morning wakefulness. This finding aligns with previous research [81–83] and highlights the relevance of considering academic environment in relation to sleep quality. The relationship between academic stress and sleep quality may be bidirectional: insufficient sleep can exacerbate stress responses and impair academic performance, while academic demands can delay bedtimes and increase arousal [59]. Consistent with biopsychosocial theory, these stress-related mechanisms operate within adolescents’ social environments, suggesting potential pathways linking perceived demands, affective states, and sleep patterns. Future research could explore the effect of including stress management strategies – such as relaxation techniques, emotional regulation training, and time management – in school curricula.

Several other associations observed in prior research, such as those with bullying victimization, physical activity, substance use or specific types of media use. While these factors may still contribute to individual differences in sleep, their effects were small and inconsistent.

Overall, this study highlights the central importance of sleep duration, gender, and the social context for adolescent sleep health. Interventions promoting sufficient sleep and addressing psychosocial stress – particularly academic pressure – should be prioritized. Strengthening family support and integrating stress management and sleep education into school programs may enhance adolescents’ overall well-being. Integrating sleep health into national adolescent health promotion strategies would thus align with contemporary public health frameworks recognizing sleep as a vital pillar of physical, mental, and social health.

### Limitations

Several limitations should be noted. First, the cross-sectional design of the study limits the ability to draw causal inferences between the bio-psycho-socio-behavioral factors and sleep quality. Longitudinal studies are needed to better understand the temporal and potentially bidirectional relationships among psychosocial stressors, sleep behaviors, and adolescent well-being.

Second, the study relied on self-reported measures of sleep quality and related behaviors, which may introduce recall bias or subjective interpretation of sleep experiences. While self-report tools such as the short Adolescent Sleep Wake Scale (ASWS) are widely used and well-validated for assessing perceived sleep patterns and psychological dimensions of sleep, they do not capture physiological aspects such as sleep architecture or sleep efficiency measured through biological markers. Future research should consider incorporating objective methods, such as actigraphy or polysomnography, to enhance measurement precision.

Third, although the study provides valuable insights into the sleep quality of Czech adolescents, a population with notably short average sleep durations, caution is warranted when generalizing the findings to adolescents in other cultural or geographic contexts. Differences in educational systems, family dynamics, or societal stressors may influence the generalizability of the observed associations.

Finally, while the study included a broad set of variables, some potentially relevant factors – such as chronotype, parental sleep habits, or pubertal development – were not assessed. Future research could expand on these dimensions to provide a more comprehensive picture of adolescent sleep determinants.

### Implications for public health and practice

Integrating these multilevel findings reinforces the view that adolescent sleep quality is a product of dynamic interactions between individual biology, psychosocial functioning, and contextual environments such as school and family systems. In line with the biopsychosocial model, effective interventions should therefore combine behavioral education (e.g., stress management, sleep hygiene) with structural and relational supports that address contextual stress.

Given sleep’s foundational role in adolescent development, interventions that target academic stress, screen time, and sleep hygiene are essential. Schools and families can collaborate on educational programs to promote healthy sleep behaviors and support routines. Tailored support for girls may reduce disparities in sleep health. Exploring individual chronotypes and allowing more flexible school schedules could further optimize sleep outcomes.

Parenting practices also play a critical role in shaping sleep behaviors. Programs that encourage autonomy-supportive parenting can facilitate adolescents’ transition to independent, healthy sleep routines. Finally, school-based interventions incorporating mindfulness, cognitive-behavioral skills, and emotional literacy may enhance resilience to stress and support better sleep outcomes.

## Conclusion

In summary, this study contributes to our understanding of adolescent sleep by highlighting the multifaceted influences of gender, academic pressure, health status, and social support in shaping sleep quality. The findings emphasize the importance of a holistic, context-sensitive approach to promoting healthy sleep habits – one that acknowledges not only behavioral factors, but also the emotional and environmental contexts in which adolescents live. Implementing targeted interventions that reduce stress, strengthen family and school support, and raise awareness about sleep hygiene may help mitigate the negative consequences of poor sleep quality in this vulnerable population and potentially support their cognitive, emotional, and physical development.

Although no single independent variable in our models reached the 0.5 SD threshold to be considered a large clinical effect [51], the factors operate additively in the multivariate model. Consequently, adolescents with multiple positive or negative factors may experience a cumulative effect that reaches or surpasses the threshold for clinically meaningful effect. This cumulative-burden perspective aligns with bio-psycho-social-behavioral models and suggests that population-level interventions targeting multiple modest-risk factors simultaneously (e.g., reducing academic pressure + increasing family support + limiting screen time) could produce clinically meaningful improvements in sleep quality, even if each change appears small in isolation.

## Supplementary Information

Supplementary Material 1.

## Data Availability

The datasets generated and/or analyzed during the current study are available in the OSF repository, 10.17605/OSF.IO/MURY3.

## Abbreviations

ASWS: Adolescent Sleep Wake Scale
CDC: Centers for Disease Control and Prevention
CI: Confidence Interval
e.g.: exempli gratia
ERDF: European Regional Development Fund
ESF: European Social Fund
FAS: Family Affluence Scale
HBSC: Health Behavior in School–aged Children
MAR: Missing At Random
MICE: Multiple Imputation by Chained Equations
MID: Minimally Important Difference
MVPA: Moderate to Vigorous Physical Activity
No.: number
OSF: Open Science Framework
ORCID: Open Researcher and Contributor ID
PSQI: Pittsburgh Sleep Quality Index
R: statistical programming language and environment
SD: Standard Deviation
SES: Socio–Economic Status
Stdβ: fully standardized beta coefficient
VIF: variation inflation factors
WHO: World Health Organization
