# Supplementary material for: Beyond counting sheep: social and behavioral determinants of adolescent sleep quality in the Czech Republic
Source: BMC Public Health. 2026 Jan 30;26:732. doi: 10.1186/s12889-025-26052-2 (PMC12930969; doi:10.1186/s12889-025-26052-2)
Supplement: Supplementary file 1 — Supplementary Material 1. [file 12889_2025_26052_MOESM1_ESM.docx]

Supplementary Figure S1: Flow diagram of participant exclusion

Total respondents in Czech HBSC 2022 dataset

**N=14,588**

The final analytical sample comprised

**N = 4,508 adolescents**.

Excluded **N =** **589** respondents

(incomplete or invalid responses)

Students from 7^th^ and 9 ^th^ grades, who completed a version of the HBSC questionnaire including items on sleep quality.

**N = 5097**

Excluded **N =** **9,491** respondents

(ineligible age or no sleep quality questionnaire)

Supplementary Figure S2: Hypothesized relations between the dependent and independent variables, organized according to the bio-psycho-social-behavioral framework.

**Sociodemographic
& Biological Variables**

**Health Behaviors**

| - Length of sleep - Alcohol consumption - Smoking - Energy drink consumption - Physical activity at least 60 min - Electronic gaming - Social media communication] - Watching TV/videos - Internet browsing |
| --- |
|  |
|  |

| - Academic pressure - Bullying victimization |
| --- |
| - Cyberbullying victimization |
|  |

**Psychological Variables & Stress**

**SLEEP QUALITY**

| - Sex - Age |
| --- |
| - SES - Health |
|  |

| - Family Support |
| --- |
| - Friend Support |
| - Teacher Support |
| - Student/classmate Support |

**Social Environment Variables**

Supplementary Table S3: Summary of the regression analysis on complete cases (pairwise deletion of missing data); Overall sleep quality and its related subscales, i.e. bedtime behaviors, sleep efficiency, and morning wakefulness (dependent variables) in association with biological, psychological, social, and behavioral factors (independent variables); Health Behaviour in School-aged Children study, Czech Republic, 2022.

|  | Overall Sleep Quality | | | |  | Bedtime Behaviors | | | |
| --- | --- | --- | --- | --- | --- | --- | --- | --- | --- |
| Independent variables | b (95% CI) | β (95% CI) | p-value | VIF |  | b (95% CI) | β (95% CI) | p-value | VIF |
| *Socio-demographic and biological variables* |  |  |  |  |  |  |  |  |  |
| Sex [Girls vs Boys] | -2.27 (-2.80, -1.74) |  | <0.001 | 1.57 |  | -0.11 (-0.33, 0.12) |  | 0.348 | 1.47 |
| Age [15 vs 13 years] | 1.27 (0.75, 1.79) |  | <0.001 | 1.23 |  | 0.49 (0.30, 0.69) |  | <0.001 | 1.17 |
| FAS | 0.00 (-0.10, 0.10) | 0.00 (-0.23, 0.23) | 0.984 | 1.12 |  | 0.00 (-0.04, 0.04) | 0.00 (-0.10, 0.09) | 0.951 | 1.17 |
| Health | 1.70 (1.31, 2.08) | 1.23 (0.95, 1.52) | <0.001 | 1.52 |  | 0.22 (0.05, 0.39) | 0.16 (0.04, 0.28) | 0.011 | 1.35 |
| *Stressors* |  |  |  |  |  |  |  |  |  |
| Bullying victimization | -0.44 (-0.77, -0.10) | -0.38 (-0.67, -0.09) | 0.010 | 1.36 |  | -0.01 (-0.15, 0.14) | -0.01 (-0.13, 0.12) | 0.911 | 1.26 |
| Cyberbullying victimization | -0.26 (-0.70, 0.18) | -0.15 (-0.41, 0.11) | 0.247 | 1.22 |  | -0.02 (-0.21, 0.17) | -0.01 (-0.13, 0.10) | 0.842 | 1.14 |
| Academic pressure | -1.80 (-2.06, -1.53) | -1.71 (-1.96, -1.46) | <0.001 | 1.47 |  | -0.25 (-0.37, -0.14) | -0.24 (-0.35, -0.13) | <0.001 | 1.32 |
| *Social environment* |  |  |  |  |  |  |  |  |  |
| Family Support [1-7] | 0.83 (0.65, 1.01) | 1.43 (1.12, 1.73) | <0.001 | 1.65 |  | 0.20 (0.13, 0.27) | 0.34 (0.22, 0.46) | <0.001 | 1.67 |
| Friend Support [1-7] | 0.06 (-0.11, 0.22) | 0.09 (-0.18, 0.37) | 0.514 | 1.31 |  | -0.01 (-0.07, 0.06) | -0.02 (-0.12, 0.09) | 0.767 | 1.45 |
| Teacher Support [0-4] | 0.46 (0.17, 0.74) | 0.42 (0.16, 0.68) | 0.002 | 1.21 |  | 0.08 (-0.03, 0.20) | 0.08 (-0.03, 0.19) | 0.161 | 1.34 |
| Student Support [0-4] | 0.69 (0.34, 1.05) | 0.64 (0.32, 0.97) | <0.001 | 1.64 |  | 0.09 (-0.04, 0.22) | 0.08 (-0.04, 0.21) | 0.188 | 1.49 |
| *Health behaviors* |  |  |  |  |  |  |  |  |  |
| Length of sleep [hours] | 1.73 (1.45, 2.00) | 2.00 (1.69, 2.32) | <0.001 | 1.52 |  | 0.88 (0.78, 0.98) | 1.03 (0.91, 1.14) | <0.001 | 1.49 |
| Alcohol | -0.37 (-0.62, -0.11) | -0.41 (-0.70, -0.13) | 0.005 | 1.27 |  | -0.14 (-0.24, -0.04) | -0.16 (-0.27, -0.04) | 0.007 | 1.32 |
| Smoking | 0.01 (-0.25, 0.26) | 0.01 (-0.29, 0.30) | 0.960 | 1.22 |  | 0.11 (0.02, 0.20) | 0.13 (0.02, 0.23) | 0.017 | 1.37 |
| Energy drink consumption | -0.47 (-0.67, -0.26) | -0.74 (-1.06, -0.41) | <0.001 | 1.51 |  | -0.06 (-0.14, 0.02) | -0.10 (-0.23, 0.03) | 0.138 | 1.41 |
| Physical activity at least 60 min [1-7 days] | -0.01 (-0.13, 0.11) | -0.02 (-0.27, 0.23) | 0.868 | 1.23 |  | 0.04 (-0.02, 0.09) | 0.07 (-0.03, 0.18) | 0.176 | 1.19 |
| Electronic gaming [hours] | -0.36 (-0.49, -0.22) | -0.76 (-1.04, -0.48) | <0.001 | 1.53 |  | -0.19 (-0.25, -0.14) | -0.41 (-0.52, -0.29) | <0.001 | 1.37 |
| Social media communication [hours] | -0.19 (-0.32, -0.05) | -0.41 (-0.70, -0.12) | 0.006 | 1.44 |  | -0.08 (-0.14, -0.03) | -0.18 (-0.30, -0.07) | 0.002 | 1.57 |
| Watching TV/videos [hours] | -0.23 (-0.40, -0.07) | -0.42 (-0.71, -0.13) | 0.005 | 1.38 |  | -0.11 (-0.17, -0.04) | -0.19 (-0.30, -0.07) | 0.001 | 1.33 |
| Internet browsing [hours] | -0.14 (-0.35, 0.06) | -0.22 (-0.55, 0.10) | 0.177 | 1.36 |  | 0.05 (-0.02, 0.12) | 0.08 (-0.04, 0.19) | 0.178 | 1.22 |
| R^2^ | 0.404 | | | |  | 0.227 | | |  |
| Notes. Continuous independent variables were mean centered and standardized to compute b and β coefficients, respectively. For categorical variables, only b coefficients are shown, as standardization is not applicable. The variation inflation factors (VIF) ≤ 1.76 for all variables in all models, i.e. no multicollinearity was detected. | | | | | | | | |  |

Supplementary Table S3 (continued)

|  | Sleep Efficiency | | | |  | Morning Wakefulness | | | |
| --- | --- | --- | --- | --- | --- | --- | --- | --- | --- |
| Independent variables | b (95% CI) | β (95% CI) | p-value | VIF |  | b (95% CI) | β (95% CI) | p-value | VIF |
| *Socio-demographic and biological variables* |  |  |  |  |  |  |  |  |  |
| Sex [Girls vs Boys] | -1.42 (-1.81, -1.03) |  | <0.001 | 1.50 |  | -0.75 (-0.95, -0.54) |  | <0.001 | 1.73 |
| Age [15 vs 13 years] | 0.86 (0.50, 1.21) |  | <0.001 | 1.20 |  | -0.08 (-0.25, 0.10) |  | 0.402 | 1.32 |
| FAS | 0.05 (-0.02, 0.12) | 0.11 (-0.05, 0.28) | 0.183 | 1.12 |  | -0.05 (-0.08, -0.02) | -0.11 (-0.19, -0.04) | 0.005 | 1.19 |
| Health | 0.99 (0.74, 1.25) | 0.72 (0.54, 0.91) | <0.001 | 1.44 |  | 0.48 (0.35, 0.61) | 0.35 (0.26, 0.45) | <0.001 | 1.64 |
| *Stressors* |  |  |  |  |  |  |  |  |  |
| Bullying victimization | -0.49 (-0.74, -0.25) | -0.43 (-0.65, -0.22) | <0.001 | 1.26 |  | 0.06 (-0.04, 0.17) | 0.06 (-0.04, 0.15) | 0.226 | 1.45 |
| Cyberbullying victimization | -0.18 (-0.52, 0.16) | -0.11 (-0.31, 0.10) | 0.304 | 1.26 |  | -0.06 (-0.20, 0.08) | -0.03 (-0.12, 0.05) | 0.424 | 1.41 |
| Academic pressure | -1.07 (-1.24, -0.89) | -1.01 (-1.18, -0.84) | <0.001 | 1.31 |  | -0.48 (-0.58, -0.38) | -0.46 (-0.55, -0.36) | <0.001 | 1.38 |
| *Social environment* |  |  |  |  |  |  |  |  |  |
| Family Support [1-7] | 0.37 (0.24, 0.49) | 0.63 (0.41, 0.84) | <0.001 | 1.58 |  | 0.27 (0.21, 0.32) | 0.46 (0.36, 0.55) | <0.001 | 1.76 |
| Friend Support [1-7] | -0.02 (-0.13, 0.10) | -0.03 (-0.22, 0.17) | 0.803 | 1.31 |  | 0.08 (0.02, 0.14) | 0.13 (0.04, 0.23) | 0.005 | 1.40 |
| Teacher Support [0-4] | 0.02 (-0.20, 0.24) | 0.02 (-0.18, 0.22) | 0.855 | 1.22 |  | 0.35 (0.24, 0.46) | 0.32 (0.22, 0.42) | <0.001 | 1.43 |
| Student Support [0-4] | 0.32 (0.08, 0.57) | 0.30 (0.07, 0.53) | 0.011 | 1.63 |  | 0.28 (0.18, 0.39) | 0.26 (0.17, 0.36) | <0.001 | 1.51 |
| *Health behaviors* |  |  |  |  |  |  |  |  |  |
| Length of sleep [hours] | 0.62 (0.42, 0.82) | 0.72 (0.49, 0.96) | <0.001 | 1.51 |  | 0.22 (0.13, 0.31) | 0.25 (0.15, 0.36) | <0.001 | 1.66 |
| Alcohol | -0.19 (-0.38, 0.00) | -0.21 (-0.42, 0.00) | 0.048 | 1.26 |  | -0.04 (-0.12, 0.05) | -0.04 (-0.14, 0.05) | 0.369 | 1.65 |
| Smoking | -0.10 (-0.31, 0.11) | -0.11 (-0.36, 0.13) | 0.369 | 1.29 |  | -0.01 (-0.08, 0.07) | -0.01 (-0.10, 0.08) | 0.835 | 1.70 |
| Energy drink consumption | -0.33 (-0.48, -0.19) | -0.53 (-0.76, -0.29) | <0.001 | 1.56 |  | -0.07 (-0.14, -0.01) | -0.11 (-0.21, -0.01) | 0.030 | 1.39 |
| Physical activity at least 60 min [1-7 days] | -0.13 (-0.21, -0.04) | -0.25 (-0.43, -0.07) | 0.006 | 1.28 |  | 0.08 (0.04, 0.12) | 0.16 (0.07, 0.24) | <0.001 | 1.36 |
| Electronic gaming [hours] | -0.10 (-0.20, 0.00) | -0.22 (-0.43, 0.00) | 0.048 | 1.67 |  | -0.06 (-0.11, -0.02) | -0.14 (-0.23, -0.05) | 0.003 | 1.44 |
| Social media communication [hours] | 0.02 (-0.08, 0.12) | 0.05 (-0.17, 0.26) | 0.663 | 1.50 |  | -0.13 (-0.17, -0.08) | -0.27 (-0.37, -0.18) | <0.001 | 1.45 |
| Watching TV/videos [hours] | -0.10 (-0.22, 0.02) | -0.18 (-0.39, 0.04) | 0.108 | 1.61 |  | -0.03 (-0.08, 0.02) | -0.06 (-0.15, 0.04) | 0.234 | 1.42 |
| Internet browsing [hours] | -0.33 (-0.48, -0.17) | -0.52 (-0.76, -0.27) | <0.001 | 1.37 |  | 0.14 (0.08, 0.20) | 0.22 (0.12, 0.31) | <0.001 | 1.29 |
| R^2^ | 0.260 | | | |  | 0.315 | | |  |
| Notes. Continuous independent variables were mean centered and standardized to compute b and β coefficients, respectively. For categorical variables, only b coefficients are shown, as standardization is not applicable. The variation inflation factors (VIF) ≤ 1.76 for all variables in all models, i.e. no multicollinearity was detected. | | | | | | | | |  |

Supplementary Table S4: Summary of fully standardized regression analysis; Overall sleep quality and its related subscales, i.e. bedtime behaviors, sleep efficiency, and morning wakefulness (dependent variables) in association with biological, psychological, social, and behavioral factors (independent variables); Health Behaviour in School-aged Children study, Czech Republic, 2022.

|  | Overall Sleep Quality | |  | Bedtime Behaviors | |  | Sleep Efficiency | |  | Morning Wakefulness | |
| --- | --- | --- | --- | --- | --- | --- | --- | --- | --- | --- | --- |
| Independent variables | Stdβ (95% CI) | p-value |  | Stdβ (95% CI) | p-value |  | Stdβ (95% CI) | p-value |  | Stdβ (95% CI) | p-value |
| *Socio-demographic variables* |  |  |  |  |  |  |  |  |  |  |  |
| Sex [Girls vs Boys] | -0.24 (-0.30, -0.18) | <0.001 |  | -0.04 (-0.10, 0.02) | 0.215 |  | -0.23 (-0.30, -0.17) | <0.001 |  | -0.23 (-0.30, -0.17) | <0.001 |
| Age [15 vs 13 years] | 0.17 (0.11, 0.23) | <0.001 |  | 0.16 (0.10, 0.21) | <0.001 |  | 0.19 (0.13, 0.25) | <0.001 |  | -0.04 (-0.10, 0.02) | 0.229 |
| FAS | 0.00 (-0.02, 0.03) | 0.870 |  | 0.00 (-0.03, 0.03) | 0.977 |  | 0.02 (-0.01, 0.04) | 0.268 |  | -0.03 (-0.05, 0.00) | 0.077 |
| Health | 0.15 (0.12, 0.18) | <0.001 |  | 0.07 (0.03, 0.10) | <0.001 |  | 0.13 (0.10, 0.16) | <0.001 |  | 0.13 (0.10, 0.16) | <0.001 |
| *Stressors* |  |  |  |  |  |  |  |  |  |  |  |
| Bullying victimization | -0.03 (-0.06, 0.00) | 0.031 |  | 0.00 (-0.03, 0.04) | 0.846 |  | -0.06 (-0.09, -0.02) | 0.001 |  | 0.02 (-0.01, 0.04) | 0.265 |
| Cyberbullying victimization | -0.01 (-0.04, 0.01) | 0.306 |  | 0.00 (-0.03, 0.03) | 0.890 |  | -0.01 (-0.04, 0.02) | 0.342 |  | -0.01 (-0.03, 0.02) | 0.483 |
| Academic pressure | -0.19 (-0.22, -0.17) | <0.001 |  | -0.07 (-0.11, -0.04) | <0.001 |  | -0.18 (-0.21, -0.15) | <0.001 |  | -0.16 (-0.19, -0.12) | <0.001 |
| *Social environment* |  |  |  |  |  |  |  |  |  |  |  |
| Family Support [1-7] | 0.15 (0.12, 0.18) | <0.001 |  | 0.10 (0.06, 0.13) | <0.001 |  | 0.11 (0.07, 0.14) | <0.001 |  | 0.14 (0.11, 0.18) | <0.001 |
| Friend Support [1-7] | 0.01 (-0.02, 0.04) | 0.555 |  | -0.01 (-0.04, 0.02) | 0.451 |  | 0.00 (-0.03, 0.03) | 0.940 |  | 0.04 (0.01, 0.07) | 0.004 |
| Teacher Support [0-4] | 0.05 (0.02, 0.08) | <0.001 |  | 0.03 (0.00, 0.06) | 0.093 |  | 0.00 (-0.03, 0.03) | 0.946 |  | 0.12 (0.09, 0.16) | <0.001 |
| Student Support [0-4] | 0.07 (0.04, 0.11) | <0.001 |  | 0.02 (-0.01, 0.06) | 0.173 |  | 0.05 (0.02, 0.09) | 0.006 |  | 0.09 (0.06, 0.12) | <0.001 |
| *Health behaviors* |  |  |  |  |  |  |  |  |  |  |  |
| Length of sleep [hours] | 0.21 (0.18, 0.24) | <0.001 |  | 0.28 (0.24, 0.31) | <0.001 |  | 0.13 (0.09, 0.16) | <0.001 |  | 0.08 (0.05, 0.11) | <0.001 |
| Alcohol | -0.05 (-0.08, -0.02) | 0.001 |  | -0.04 (-0.08, -0.01) | 0.006 |  | -0.04 (-0.08, -0.01) | 0.010 |  | -0.01 (-0.04, 0.02) | 0.524 |
| Smoking | 0.01 (-0.02, 0.04) | 0.404 |  | 0.05 (0.02, 0.08) | 0.003 |  | -0.01 (-0.05, 0.03) | 0.599 |  | 0.01 (-0.02, 0.03) | 0.705 |
| Energy drink consumption | -0.09 (-0.12, -0.05) | <0.001 |  | -0.03 (-0.07, 0.00) | 0.067 |  | -0.10 (-0.14, -0.07) | <0.001 |  | -0.02 (-0.05, 0.01) | 0.153 |
| Physical activity at least 60 min [1-7 days] | 0.01 (-0.02, 0.03) | 0.537 |  | 0.02 (-0.01, 0.05) | 0.187 |  | -0.03 (-0.06, 0.00) | 0.043 |  | 0.07 (0.04, 0.09) | <0.001 |
| Electronic gaming [hours] | -0.08 (-0.11, -0.04) | <0.001 |  | -0.12 (-0.15, -0.08) | <0.001 |  | -0.03 (-0.07, 0.00) | 0.070 |  | -0.04 (-0.07, -0.01) | 0.017 |
| Social media communication [hours] | -0.06 (-0.09, -0.03) | <0.001 |  | -0.06 (-0.09, -0.03) | 0.001 |  | 0.00 (-0.04, 0.03) | 0.843 |  | -0.10 (-0.13, -0.07) | <0.001 |
| Watching TV/videos [hours] | -0.03 (-0.06, 0.00) | 0.039 |  | -0.05 (-0.08, -0.02) | 0.003 |  | -0.01 (-0.05, 0.02) | 0.432 |  | -0.02 (-0.05, 0.01) | 0.285 |
| Internet browsing [hours] | -0.03 (-0.06, 0.01) | 0.123 |  | 0.03 (0.00, 0.06) | 0.068 |  | -0.10 (-0.14, -0.06) | <0.001 |  | 0.08 (0.05, 0.11) | <0.001 |
| Notes. All continuous variables (dependent and independent) were standardized to compute β coefficients. | | | | | |  |  |  |  |  |  |
